# Supplementary material for: Time-series transcriptome analysis identified differentially expressed genes in broiler chicken infected with mixed Eimeria species
Source: Front Genet. 2022 Aug 8;13:886781. doi: 10.3389/fgene.2022.886781 (PMC9393255; doi:10.3389/fgene.2022.886781)
Supplement: Supplementary file 2 [file DataSheet1.ZIP › 4dpi_KEGG.Gsea.1625100298584/KEGG_TOLL_LIKE_RECEPTOR_SIGNALING_PATHWAY.html]

Details for gene set KEGG\_TOLL\_LIKE\_RECEPTOR\_SIGNALING\_PATHWAY[GSEA]

|  || Dataset | TMM\_4dpi\_gct\_format\_4dpi\_gct\_format.Class\_4dpi.cls #PC\_versus\_NC.Class\_4dpi.cls #PC\_versus\_NC\_repos |
| Phenotype | Class\_4dpi.cls#PC\_versus\_NC\_repos |
| Upregulated in class | 1 |
| GeneSet | KEGG\_TOLL\_LIKE\_RECEPTOR\_SIGNALING\_PATHWAY |
| Enrichment Score (ES) | 0.39372265 |
| Normalized Enrichment Score (NES) | 1.4461197 |
| Nominal p-value | 0.031438127 |
| FDR q-value | 0.33061236 |
| FWER p-Value | 0.9964 |
Table: GSEA Results Summary

  

Fig 1: Enrichment plot: KEGG\_TOLL\_LIKE\_RECEPTOR\_SIGNALING\_PATHWAY      
 Profile of the Running ES Score & Positions of GeneSet Members on the Rank Ordered List

  

| SYMBOL | TITLE | RANK IN GENE LIST | RANK METRIC SCORE | RUNNING ES | CORE ENRICHMENT || 1 | TLR3 | na | 40 | 2.065 | 0.0712 | Yes |
| 2 | STAT1 | na | 78 | 1.743 | 0.1309 | Yes |
| 3 | IRF7 | na | 85 | 1.706 | 0.1920 | Yes |
| 4 | MYD88 | na | 304 | 1.174 | 0.2161 | Yes |
| 5 | CCL5 | na | 438 | 1.026 | 0.2420 | Yes |
| 6 | IKBKE | na | 505 | 0.959 | 0.2711 | Yes |
| 7 | PIK3CA | na | 515 | 0.948 | 0.3045 | Yes |
| 8 | IFNAR1 | na | 640 | 0.855 | 0.3250 | Yes |
| 9 | MAPK13 | na | 870 | 0.727 | 0.3320 | Yes |
| 10 | JUN | na | 912 | 0.705 | 0.3540 | Yes |
| 11 | IFNAR2 | na | 1101 | 0.637 | 0.3612 | Yes |
| 12 | FOS | na | 1216 | 0.599 | 0.3733 | Yes |
| 13 | MAP3K8 | na | 1229 | 0.594 | 0.3937 | Yes |
| 14 | RAC1 | na | 1737 | 0.468 | 0.3682 | No |
| 15 | CCL4 | na | 1945 | 0.433 | 0.3664 | No |
| 16 | MAPK12 | na | 2084 | 0.408 | 0.3696 | No |
| 17 | CASP8 | na | 2138 | 0.398 | 0.3795 | No |
| 18 | AKT3 | na | 2430 | 0.357 | 0.3680 | No |
| 19 | PIK3CB | na | 2649 | 0.329 | 0.3616 | No |
| 20 | MAPK9 | na | 2786 | 0.310 | 0.3614 | No |
| 21 | FADD | na | 2797 | 0.309 | 0.3718 | No |
| 22 | TIRAP | na | 3000 | 0.282 | 0.3650 | No |
| 23 | IKBKB | na | 3506 | 0.220 | 0.3307 | No |
| 24 | TAB2 | na | 3587 | 0.208 | 0.3315 | No |
| 25 | TRAF6 | na | 3778 | 0.190 | 0.3224 | No |
| 26 | CTSK | na | 4067 | 0.162 | 0.3041 | No |
| 27 | MAPK14 | na | 4107 | 0.159 | 0.3066 | No |
| 28 | RIPK1 | na | 4121 | 0.158 | 0.3112 | No |
| 29 | MAP2K2 | na | 4498 | 0.124 | 0.2842 | No |
| 30 | MAPK3 | na | 4570 | 0.119 | 0.2826 | No |
| 31 | CD80 | na | 4648 | 0.111 | 0.2801 | No |
| 32 | MAP2K1 | na | 4692 | 0.107 | 0.2804 | No |
| 33 | TBK1 | na | 4986 | 0.079 | 0.2587 | No |
| 34 | CD14 | na | 5067 | 0.073 | 0.2546 | No |
| 35 | MAP2K4 | na | 5132 | 0.067 | 0.2517 | No |
| 36 | IRAK4 | na | 5151 | 0.064 | 0.2525 | No |
| 37 | TICAM1 | na | 5280 | 0.056 | 0.2438 | No |
| 38 | AKT1 | na | 5330 | 0.052 | 0.2416 | No |
| 39 | TRAF3 | na | 5463 | 0.040 | 0.2320 | No |
| 40 | NFKBIA | na | 5648 | 0.021 | 0.2173 | No |
| 41 | MAPK10 | na | 5730 | 0.014 | 0.2110 | No |
| 42 | MAP2K3 | na | 5841 | 0.006 | 0.2021 | No |
| 43 | RELA | na | 5979 | -0.005 | 0.1908 | No |
| 44 | PIK3R2 | na | 5995 | -0.006 | 0.1897 | No |
| 45 | TLR4 | na | 6033 | -0.009 | 0.1869 | No |
| 46 | MAP3K7 | na | 6443 | -0.042 | 0.1542 | No |
| 47 | PIK3R1 | na | 6590 | -0.054 | 0.1439 | No |
| 48 | NFKB1 | na | 6765 | -0.068 | 0.1318 | No |
| 49 | MAPK8 | na | 6831 | -0.073 | 0.1290 | No |
| 50 | TOLLIP | na | 7053 | -0.094 | 0.1139 | No |
| 51 | LY96 | na | 7083 | -0.097 | 0.1149 | No |
| 52 | MAP2K6 | na | 7919 | -0.172 | 0.0512 | No |
| 53 | PIK3CG | na | 8485 | -0.227 | 0.0121 | No |
| 54 | CD86 | na | 8523 | -0.232 | 0.0174 | No |
| 55 | PIK3R5 | na | 9216 | -0.311 | -0.0294 | No |
| 56 | CHUK | na | 9400 | -0.336 | -0.0326 | No |
| 57 | TAB1 | na | 9611 | -0.363 | -0.0370 | No |
| 58 | LBP | na | 9889 | -0.396 | -0.0460 | No |
| 59 | PIK3CD | na | 10148 | -0.437 | -0.0518 | No |
| 60 | MAPK11 | na | 10200 | -0.444 | -0.0400 | No |
| 61 | TLR7 | na | 10620 | -0.523 | -0.0563 | No |
| 62 | TLR5 | na | 10718 | -0.542 | -0.0448 | No |
| 63 | CD40 | na | 10769 | -0.552 | -0.0291 | No |
| 64 | IL1B | na | 11033 | -0.617 | -0.0289 | No |
| 65 | IRF5 | na | 11267 | -0.688 | -0.0235 | No |
| 66 | PIK3R3 | na | 11670 | -0.936 | -0.0234 | No |
| 67 | SPP1 | na | 11930 | -1.436 | 0.0067 | No |
Table: GSEA details [plain text format]

  

Fig 2: KEGG\_TOLL\_LIKE\_RECEPTOR\_SIGNALING\_PATHWAY      
 Blue-Pink O' Gram in the Space of the Analyzed GeneSet

  

Fig 3: KEGG\_TOLL\_LIKE\_RECEPTOR\_SIGNALING\_PATHWAY: Random ES distribution      
 Gene set null distribution of ES for **KEGG\_TOLL\_LIKE\_RECEPTOR\_SIGNALING\_PATHWAY**
